# Characterization and risk assessment of novel SXT/R391 integrative and conjugative elements with multidrug resistance in *Proteus mirabilis* isolated from China, 2018–2020

**DOI:** 10.1128/spectrum.01209-23

**Published:** 2024-01-10

**Authors:** Yun Han, Yu-Feng Gao, He-ting Xu, Jin-Peng Li, Chao Li, Cai-Liang Song, Chang-Wei Lei, Xuan Chen, Qin Wang, Bo-Heng Ma, Hong-Ning Wang

**Affiliations:** 1 Animal Disease Prevention and Food Safety Key Laboratory of Sichuan Province, College of Life Sciences, Sichuan University, Chengdu, Sichuan, China; 2 Key Laboratory of Bio-Resource and Eco-Environment of Ministry of Education, College of Life Sciences, Sichuan University, Chengdu, Sichuan, China; Institute of Parasitology, Biology Centre, ASCR, Ceske Budejovice, Czechia

**Keywords:** *Proteus mirabilis*, multidrug resistance, SXT/R391 integrating conjugative elements, *cfr* gene

## Abstract

*Proteus mirabilis* can transfer transposons, insertion sequences, and gene cassettes to the chromosomes of other hosts through SXT/R391 integrative and conjugative elements (ICEs), significantly increasing the possibility of antibiotic resistance gene (ARG) evolution and expanding the risk of ARGs transmission among bacteria. A total of 103 strains of *P. mirabilis* were isolated from 25 farms in China from 2018 to 2020. The positive detection rate of SXT/R391 ICEs was 25.2% (26/103). All SXT/R391 ICEs positive *P. mirabilis* exhibited a high level of overall drug resistance. Conjugation experiments showed that all 26 SXT/R391 ICEs could efficiently transfer to *Escherichia coli* EC600 with a frequency of 2.0 × 10^−7^ to 6.0 × 10^−5^. The acquired ARGs, genetic structures, homology relationships, and conservation sequences of 26 (19 different subtypes) SXT/R391 ICEs were investigated by high-throughput sequencing, whole-genome typing, and phylogenetic tree construction. ICE*Pmi*ChnHBRJC2 carries *erm (42*), which have never been found within an SXT/R391 ICE in *P. mirabilis*, and ICE*Pmi*ChnSC1111 carries 19 ARGs, including clinically important *cfr*, *bla_CTX-M-65_*, and *aac(6')-Ib-cr*, making it the ICE with the most ARGs reported to date. Through genetic stability, growth curve, and competition experiments, it was found that the transconjugant of ICE*Pmi*ChnSCNNC12 did not have a significant fitness cost on the recipient bacterium EC600 and may have a higher risk of transmission and dissemination. Although the transconjugant of ICE*Pmi*ChnSCSZC20 had a relatively obvious fitness cost on EC600, long-term resistance selection pressure may improve bacterial fitness through compensatory adaptation, providing scientific evidence for risk assessment of horizontal transfer and dissemination of SXT/R391 ICEs in *P. mirabilis*.

**IMPORTANCE:**

The spread of antibiotic resistance genes (ARGs) is a major public health concern. The study investigated the prevalence and genetic diversity of integrative and conjugative elements (ICEs) in *Proteus mirabilis*, which can transfer ARGs to other hosts. The study found that all of the *P. mirabilis* strains carrying ICEs exhibited a high level of drug resistance and a higher risk of transmission and dissemination of ARGs. The analysis of novel multidrug-resistant ICEs highlighted the potential for the evolution and spread of novel resistance mechanisms. These findings emphasize the importance of monitoring the spread of ICEs carrying ARGs and the urgent need for effective strategies to combat antibiotic resistance. Understanding the genetic diversity and potential for transmission of ARGs among bacteria is crucial for developing targeted interventions to mitigate the threat of antibiotic resistance.

## INTRODUCTION

In recent years, the prevalence of antibiotic resistance among pathogenic bacteria has become a global challenge that is closely associated with high rates of morbidity and mortality in both humans and animals (1–4). This alarming trend is reflected in the increasing resistance of several important bacterial pathogens to commonly used antimicrobial methods, as well as the emergence of multidrug-resistant strains. As a result, combating bacterial infection and disease remains a serious challenge (5).

*Proteus mirabilis* is an opportunistic pathogen of great significance from the perspective of human and animal public health, and they can express a significant amount of cytotoxins, leading to epithelial cell damage (6). *P. mirabilis* is typically a member of the normal flora inhabiting the intestines of humans and animals as well as in nature (7). However, it can also cause various types of infectious diseases in the environment and is one of the most common pathogenic factors for urinary tract infections (7–14). Molecular typing studies of *P. mirabilis* have found that strains present in animals such as livestock are closely related to those present in humans and animal strains, and the resistance can be transmitted to human beings (15–17).

The acquisition of new antibiotic resistance genes (ARGs) plays an important role in the evolution of antibiotic resistance (18, 19). Various ARGs acquired from mobile genetic elements (MGEs) have been found in *P. mirabilis* (18, 20, 21). Integrative and conjugative elements (ICEs), a member of MGEs, play a critical role in promoting the genetic evolution of bacteria by transferring genes through transposons or insertion sequences and increasing the genomic plasticity of bacteria (22). Typically, ICEs consist of a recombination, conjugation, regulation, and accessory gene structure that is highly conserved (23, 24). Although ICEs usually carry genes that are associated with host adaptation, not all ICEs confer such benefits to their hosts. Recent bioinformatics analyses have indicated that some ICEs do not provide obvious benefits to the host bacterium or that such benefits have yet to be identified and validated (25). Due to their self-transferability and diverse accessory gene pool, ICEs are essential in bacterial evolution, particularly in antibiotic resistance. Studies have shown that ICEs can mediate the dissemination and spread of crucial ARGs such as *lsa(E*), *cfr*, *bla_CMY-2_*, *bla_CTX-M-65_*, *fosA3*, and others (15, 26, 27). ICEs can enhance the diversity and plasticity of bacterial gene structures at the genomic level, accelerate gene transfer among various species through conjugative transfer, and help hosts adapt to complex and changing environments.

SXT and R391 belong to the SXT/R391 family of ICEs, which were the first discovered members of this family (28–30). They share several common features such as the ability to integrate into a specific chromosomal integration site (*prfC*) in the host bacterium, to transfer horizontally between donor and recipient bacteria via conjugation, and their conserved gene structure (31, 32). After the discovery of SXT and R391, many similar ICEs with comparable structure and function were found in other countries and strains (6, 33–35). These elements have been collectively classified into the SXT/R391 ICEs family, which is the most extensively studied in the ICEberg 2.0 classification tool (15, 33, 34, 36).

The core gene region of most SXT/R391 ICEs consists of 52 core genes, forming a conserved structural backbone (32). In addition, the five hotspots (HS1-5) and five variable regions (VRI–VRV) are the most complex and studied areas of variation.

Recently, several new ICEs carrying multiple resistance genes have been identified in animal pathogens that cause *P. mirabilis* (15, 27, 37–39). Over 1,300 types of ICEs have been found in almost all *P. mirabilis* strains in China, especially in the southwestern region, and their host range has expanded beyond γ-Proteobacteria (40). These elements undergo not only vertical transmission within their host range but also horizontal transmission between different hosts via conjugation, resulting in rapid increases in the number, structure, and spread of ICEs, which have significant impacts on antimicrobial resistance.

## RESULTS

### Detection and conjugation of SXT/R391 ICEs in *P. mirabilis*

The positive detection rate of SXT/R391 ICEs in the 103 *P*. *mirabilis* strains analyzed in this study was 25.2% (26/103; Fig. S1A through D). The conjugation transfer experiment result showed that the ICEs in all 26 *P*. *mirabilis* strains could be transferred to EC600 and integrated into the 5′ end of *prfC* on the chromosome. The binding efficiency was 2.0 × 10^−7^ to 6.0 × 10^−5^ (average of three independent analyses). The transconjugants were further detected through drug resistance spectrum and *int* gene analysis, and all transconjugants were ICE positive, as shown in Fig. S1E. Details on these 26 strains suspected to carry SXT/R391 ICEs are presented in Table 1.

**TABLE 1:** Detailed information of 26 *P*. *mirabilis* strains carrying SXT/R391 ICEs

Number	Strain	Province	Isolated date	Source
1	PmHBNNC12	Hebei, China	28 June 2018	Cattle
2	PmHBNNC21	Hebei, China	27 October 2020	Cattle
3	PmHBRJC2	Hebei, China	12 October 2020	Chicken
4	PmHBRJC7	Hebei, China	22 June 2018	Chicken
5	PmHBSZC16	Hebei, China	28 June 2018	Pig
6	PmHBSZC23	Hebei, China	17 October 2020	Pig
7	PmHERJC4	Hubei, China	23 June 2018	Chicken
8	PmHERJC7	Hubei, China	16 October 2020	Chicken
9	PmSCDJC2	Sichuan, China	28 June 2018	Chicken
10	PmSCNNC12	Sichuan, China	15 October 2020	Cattle
11	PmSCNNC24	Sichuan, China	16 October 2020	Cattle
12	PmSCRJC3	Sichuan, China	10 October 2020	Chicken
13	PmSCRJC4	Sichuan, China	25 June 2018	Chicken
14	PmSCRJC5	Sichuan, China	26 June 2018	Chicken
15	PmSCRJC7	Sichuan, China	19 October 2020	Chicken
16	PmSCSZC10	Sichuan, China	22 October 2020	Pig
17	PmSCSZC11	Sichuan, China	14 October 2020	Pig
18	PmSCSZC17	Sichuan, China	20 October 2020	Pig
19	PmSCSZC20	Sichuan, China	27 June 2018	Pig
20	PmSCSZC25	Sichuan, China	21 October 2020	Pig
21	PmSCH5	Sichuan, China	18 October 2020	Human
22	PmSC1111	Sichuan, China	11 October 2020	Pig
23	PmSCBC11-9	Sichuan, China	24 June 2018	Pig
24	PmSCSN5-5	Sichuan, China	19 October 2020	Cattle
25	PmYNDJH6	Sichuan, China	28 June 2018	Chicken
26	PmNBJFZQ1	Sichuan, China	10 December 2019	Chicken

### Antimicrobial susceptibility tests

The results of antimicrobial susceptibility testing for SXT/R391-containing *P. mirabilis* are presented in Tables 2 and 3. Among the 26 strains of *P. mirabilis* bacteria, 23 out of them (96.15%) exhibited resistance to multiple drugs, which were resistant to more than three antibiotics in addition to their intrinsic resistance profiles. The largest proportion of these resistant strains (6/26, 23.07%) were resistant to eight drugs. Ciprofloxacin exhibited the highest resistance rate among all drugs, at 96.15%. The lowest resistance rates were observed for doxycycline and naphthyric acid, both at 3.85%.

**TABLE 2:** The antimicrobial susceptibility of 26 *P*. *mirabilis* strains^*a*^

Number	Strain	Resistance
1	PmHBNNC12	AMP-CTX-CRO-C-CIP-LEV-SXT-CN-FFC-FOS
2	PmHBNNC21	AMP
3	PmHBRJC2	AMP-CTX-CRO-C-CIP-LEV-SXT-CN-FFC-FOS
4	PmHBRJC7	AMP-AMC-C-CIP-TE-SXT-CN-FFC
5	PmHBSZC16	AMP-CTX-CRO-C-CIP-SXT-CN-FFC-FOS
6	PmHBSZC23	C-TE-FFC
7	PmHERJC4	AMP-CTX-CRO-C-CIP-TE-SXT-CN-FOS-FFC
8	PmHERJC7	AMP-CTX-CRO-C-CIP-LEV-SXT-CN-FFC-FOS
9	PmSCDJC2	AMP-CTX-CRO-C-SXT-CN-FOS-FFC
10	PmSCNNC12	AMP-C-TE-SXT-FFC
11	PmSCNNC24	AMP-C-FFC
12	PmSCRJC3	AMP-CTX-C-CIP-TE-SXT-CN-FFC
13	PmSCRJC4	AMP-CTX-CRO-C-TE-SXT-FFC-FOS
14	PmSCRJC5	AMP-CTX-CRO-C-CIP-LEV-SXT-CN-FFC-FOS
15	PmSCRJC7	AMP-AMC-C-TE-SXT-CN-FFC
16	PmSCSZC10	AMP-C-SXT-CN-FFC
17	PmSCSZC11	AMP-C-SXT-FFC
18	PmSCSZC17	AMP-C-TE-SXT-FFC
19	PmSCSZC20	AMP-CTX-CRO-C-TE-SXT-CN-FFC-FOS
20	PmSCSZC25	C-TE-SXT-FFC
21	PmSCH5	SXT-AMP-DO-NA-C
22	PmSC1111	AMP-C-CIP-TE-SXT-CN-FFC
23	PmSCBC11-9	AMP-CTX-C-CIP-TE-SXT-CN-FFC-FOS
24	PmSCSN5-5	AMP-C-CIP-TE-SXT-CN-FFC
25	PmYNDJH6	AMP-C-CIP-TE-SXT-CN-FFC
26	PmNBJFZQ1	AMP-CTX-C-CIP-TE-SXT-CN-FFC

^a^ SXT, compound sulfamethoxazole; AMP, ampicillin; FFC, florfenicol; CIP, ciprofloxacin; AMC, amoxycillin/clavulanic; CTX, cefotaxime; acid; CRO, cefatriaxone; C, chloramphenicol; LEV, levofloxacin; CN, gentamicin; FOS, fosfomycin; TE, tetracycline; DO, doxycycline; NA, naphthyric acid.

**TABLE 3:** The drug resistance rate of 26 *P. mirabilis* strains*^a^*

Antibiotics	R (strains)	I + S (strains)	Rate (%)
SXT	23	3	88.46%
AMP	24	2	92.31%
FFC	24	2	92.31%
CIP	13	13	50.00%
AMC	2	24	7.69%
CTX	12	14	46.15%
CRO	9	17	34.62%
C	25	1	96.15%
LEV	4	22	15.38%
CN	18	8	69.23%
FOS	9	17	34.62%
TE	14	12	53.85%
DO	1	25	3.85%
NA	1	25	3.85%

^a^ SXT, compound sulfamethoxazole; AMP, ampicillin; FFC, florfenicol; CIP, ciprofloxacin; AMC, amoxycillin/clavulanic; CTX, cefotaxime; acid; CRO, cefatriaxone; C, chloramphenicol; LEV, levofloxacin; CN, gentamicin; FOS, fosfomycin; TE, tetracycline; DO, doxycycline; NA, naphthyric acid. R, resistant; I, intermediate; S, susceptible.

### Genomic typing of multidrug-resistant *P. mirabilis*

**Fig 1:**
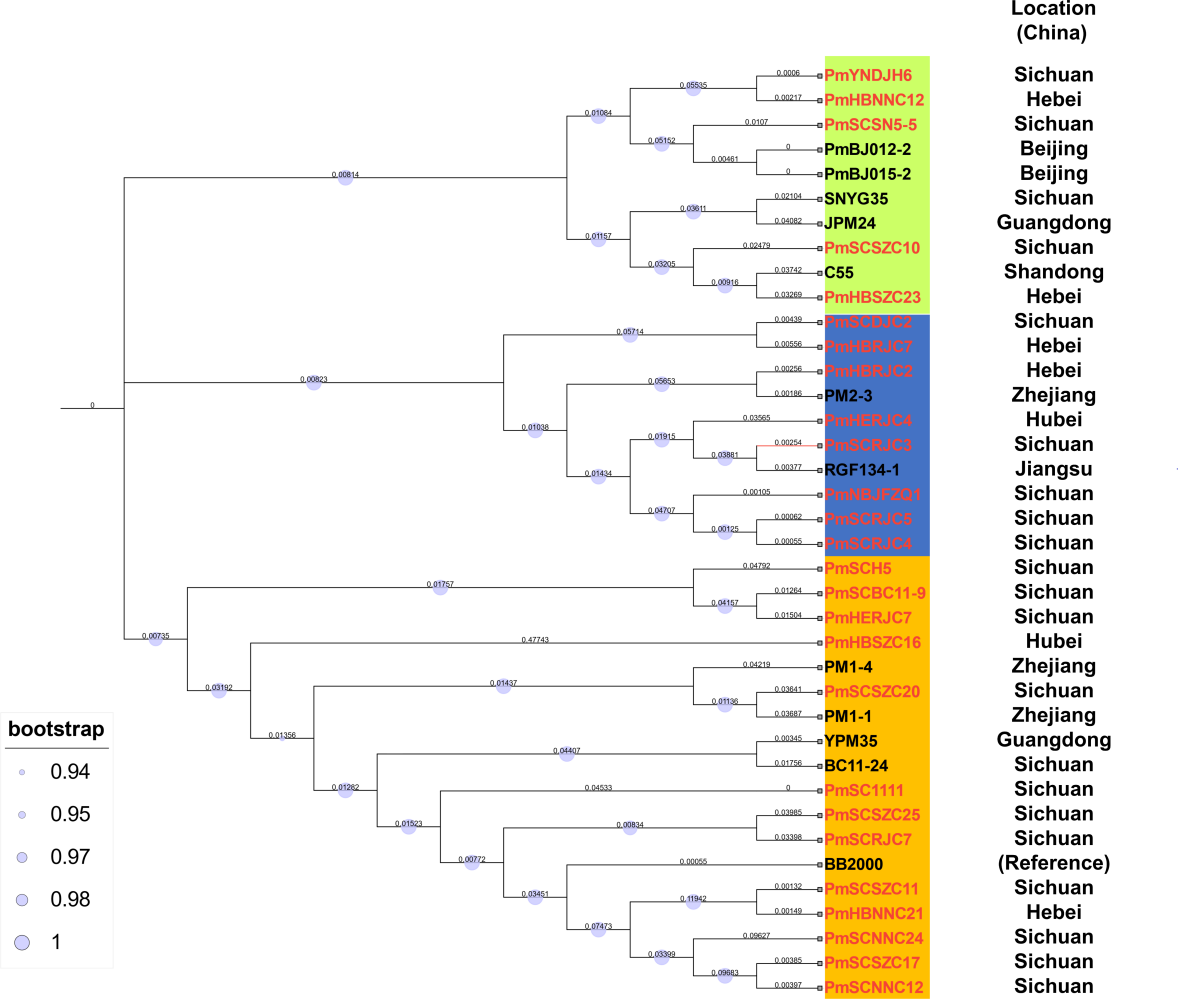
Single nucleotide polymorphism phylogenetic tree based on *P. mirabilis strains*. Bootstrap analysis was calculated from 10^3^ replicates. Bar, 0.05 substitutions per nucleotide position.

A genetic relationship based on the whole-genome was constructed for 26 *P. mirabilis* strains in this study and other isolates from different geographic locations in China (Table 1; Table S1), which revealed three distinct groups (Fig. 1). The strains isolated from three different provinces are widely dispersed on the tree, indicating their genetic diversity. In contrast, isolates from different geographic locations cluster closely within evolutionary branches. These findings suggest the potential clonal expansion of multidrug-resistant *P. mirabilis* across China.

### Genetic structure of novel multidrug-resistant SXT/R391 ICEs

**Fig 2:**
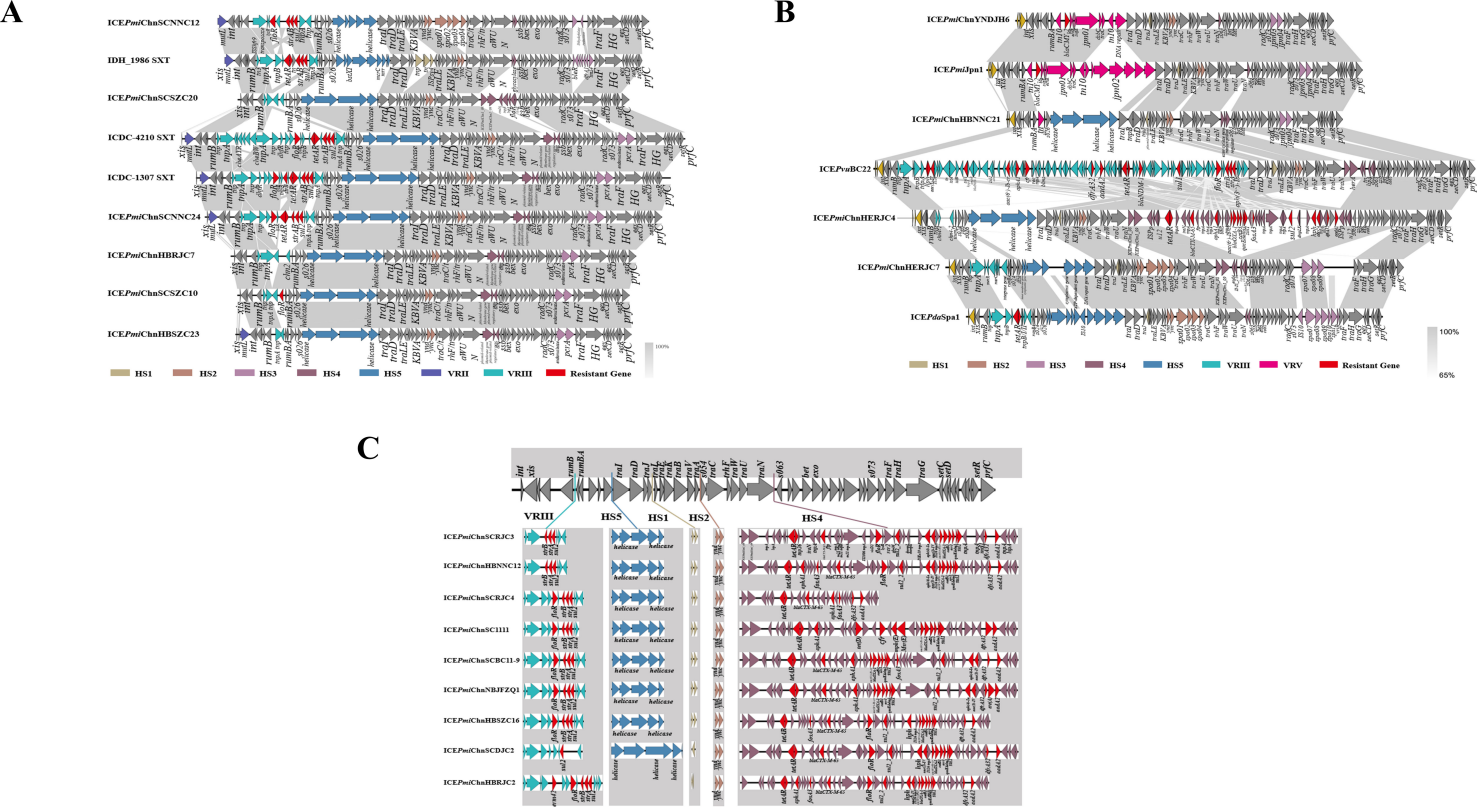
Gene structure of SXT/R391 ICEs. (**A**) Schematic diagram of gene structure of six SXT/R391 ICEs. ICE*Pmi*ChnSCNNC12, ICE*Pmi*ChnSCSZC20, ICE*Pmi*ChnSCNNC24, ICE*Pmi*ChnHBRJC7, ICE*Pmi*ChnSCSZC10, and ICE*Pmi*ChnHBSZC23 found in this study showed almost 100% nucleotide identity with from IDH_1986 SXT (MK165649), ICDC-4210 SXT (KT151662), and ICDC-1307 SXT (KJ817376), respectively. (**B**) Schematic diagram of gene structure of four SXT/R391 ICEs. ICE*Pmi*ChnSCNNC24, ICE*Pmi*ChnHBRJC7, ICE*Pmi*ChnSCSZC10, and ICE*Pmi*ChnHBSZC23 share 97%, 96%, 96%, and 95% nucleotide identity, respectively, with ICDC-1307 SXT (KJ817376). (**C**) Schematic diagram of gene structure of nine SXT/R391 ICEs. ICE*Pmi*ChnSCRJC3, ICE*Pmi*ChnHBNNC12, ICE*Pmi*ChnSCRJC4, ICE*Pmi*ChnSCRJC5, ICE*Pmi*ChnSC1111, ICE*Pmi*ChnSCBC11-9, ICE*Pmi*ChnNBJFZQ1, ICE*Pmi*ChnHBSZC16, ICE*Pmi*ChnSCDJC2, and ICE*Pmi*ChnHBRJC2 are highly similar to ICE*Pmi*ChnBCP11 (MG773277), with nucleotide similarities ranging from 83% to 96%.

In the following sections of this study, we will delve into a detailed comparative analysis of ICEs, exploring features such as Hotspots (HS) and Variable Regions (VR). Sequence analysis revealed that all 26 *P. mirabilis* strains contained SXT/R391 ICEs, with 7 of them being nearly identical, leaving 19 distinct types. The determination of the 19 different subtypes of ICEs is based on structural variations within the variable regions and hotspots of ICEs, as well as the composition of resistance genes they carry. Fig. 2A through C displays the genetic structure diagram of these 19 different SXT/R391 ICEs and their related reference ICEs. By comparing these ICEs, we aim to decipher the underlying genetic mechanisms driving their diversity, mobility, and impact on bacterial populations.

ICE*Pmi*ChnSCNNC12 and IDH_ 1986 SXT (MK165649) share 72% nucleotide identity, and the HS1 region was found to be short (Fig. 2A). HS1 is shorter in ICEPmiChnSCNNC12 (Fig. 2A). Both contain a mosA/T toxin-antitoxin system in HS2, closely related to ICEVscSpa2. ICEPmiChnSCNNC12 also has VRIII with multidrug resistance genes: *floR* (chloramphenicol), *strAB* (streptomycin), and *sul2* (sulfamethoxazole). ICE*Pmi*ChnSCSZC20 shares 85% nucleotide identity with ICDC-4210 SXT (KT151662). In HS2, two genes, *ynd* and *ync*, closely resemble ICEPmiChnHERJC7 and ICEPdaSpa1 (AJ870986). HS4 contains seven open reading frames (ORFs), matching the structure of ICEPmiChn1 (KT962845). HS5 has five ORFs, mirroring the structure of ICDC-4210 SXT (KT151662). ICE*Pmi*ChnYNDJH6 shares 97% nucleotide identity with ICE*Pmi*Jpn1 (KT894734), which was previously reported to carry *bla_CMY-2_* in Japan, Spain, and China (15, 34, 36). Both ICEs have similar VRI, HS1, HS2, HS3, and HS4 regions but differ in VRV and HS5 regions (Fig. 2B). Our study found that ICEPmiChnYNDJH6, ICEPmiChnSCH5, and ICEPmiChnSN5-5 accounted for 11.54% of *P. mirabilis* strains carrying blaCMY-2, while other studies reported higher proportions (15, 36). In summary, SXT/R391 ICE plays an important role in the transmission of *bla_CMY-2_* in *P. mirabilis*.

In strain PmHBNNC21, ICE*Pmi*ChnHBNNC21 shares 69% nucleotide identity with ICE*Pmi*Jpn1. The HS2 region carries two genes, *ynd* and *ync*, as in ICE*Pmi*Jpn1, but the surrounding gene environment of these genes is different. The strain PmHERJC4 carries ICE*Pmi*ChnHERJC4, which shares 71% nucleotide identity with ICEPvuBC22 (MH160822) reported in our previous study (38). The *chn4-2* gene, encoding β-lactamase and resistant to β-lactam antibiotics, was first found in the HS4 region of ICE*Pmi*ChnHERJC4, flanked by insertions that could also be found in the genome of *Vibrio parahaemolyticus* UCM-V493. ICE*Pmi*ChnHERJC7 identified in strain PmHERJC7 shares 71% nucleotide identity with worldwide circulating ICE*Pda*Spa1 (AJ870986). Compared with ICE*Pda*Spa1, there are only spa07, spa08, and spa09 in the HS3 region, and some gene sequences are missing, and mutations may have occurred. ICE*Pmi*ChnSCRJC3 and ICE*Pmi*ChnHBNNC12 lack one copy of the *floR* gene, while ICE*Pmi*ChnSCDJC2 has an additional copy of the *erm (42)* gene and a different genetic environment (Fig. 2C). ICE*Pmi*ChnHBRJC2 lacks the HS5 region, and two genes in the HS1 region are in opposite direction but are the same as those in ICE*Pmi*ChnBCP11. This phenomenon has not been previously reported.

The structures of ICE*Pmi*ChnSCRJC3, ICE*Pmi*ChnHBNNC12, ICE*Pmi*ChnSCRJC4, ICE*Pmi*ChnSCRJC5, ICE*Pmi*ChnSC1111, PmSCBC11-9, ICE*Pmi*ChnNBJFZQ1, ICE*Pmi*ChnHBSZC16, ICE*Pmi*ChnSCDJC2, and ICE*Pmi*ChnHBRJC2 differ from ICEP1miChnBCP in the HS4 region. These ICEs carry 15, 17, 7, 7, 15, 16, 16, 15, 15, and 17 ARGs, respectively, including *cfr*, *bla_CTX-M-65_*, and *fosA3*, *aac(6')-Ib-cr*, *dfrA32*, *ereA*, *aadA2*, *hph*, and *aacC4*. Table 4 shows the composition forms of all ARG cassettes in the HS4 region.

**TABLE 4:** Summary of drug resistance gene box in HS4 region^*a*^

ICE	Drug resistance gene box											
ICE*Pmi*ChnBCP11	*ISPpu12-tetA-tetR-IS26*	*IS903D-bla_CTX-M-65_-fip-IS26*	*IS26-aphA1-IS26*	*IS26-blmS-IS26*	*IS26-aac(6’)-Ib-cr-bla_OXA-1_-catB3-arr3-qacEdelta1-sul1-IS26*	*IS26-fosA3-orf1-orf2-orf3-IS26*	*ISEc59-hph-aacC4-IS26*	*IS26-IS26-IS26-IS26-orf-sul2-dumpL-dumpK-IS26*	*intI1-dfrA32-ereA1-aadA2-ISPpu12*	*IS26-cfr-IS26*		
ICE*Pmi*ChnSCRJC3	*ISPpu12-orf1-orf2-orf3-tetA-tetR-IS26*	★			★		★	*IS1006-virD2-floR-lysR-rcr2-glmM-sul2-IS26*	*intI1-dfrA32-aadA2-ISPpu12*			
ICE*Pmi*ChnHBNNC12	★	★	★		★	*IS26-orf1-orf2-orf3-fosA3-IS26*		*IS1006-virD2-floR-lysR-rcr2-glmM-sul2-IS26*	*intI1-dfrA32-aadA2-ISPpu12*			
ICE*Pmi*ChnSCRJC4	★	★	★			*IS26-orf1-orf2-orf3-fosA3-IS26*			*intI1-dfrA32-aadA2-ISPpu12*			
ICE*Pmi*ChnSC1111	*ISPpu12-orf1-orf2-tetA-tetR-IS26*		★	*IS26-NimC-blmS-IS26*			*IS26-aacC4-IS26*		*intI1-dfrA32-aadA2-ISPpu12*	★	*IS26-orf1-tetD-orf2-IS26*	*IS26-mphE-msrE-orf1-IS26*
ICE*Pmi*ChnSCBC11-9	★	★	★		★	*IS26-fosA3-IS26*		*IS26-orf1-sul2-IS26*	*intI1-dfrA32-aadA2-ISPpu12*			
ICE*Pmi*ChnNBJFZQ1	★	★	★		★		*★*	*IS26-orf1-sul2-IS26*	★			
ICE*Pmi*ChnHBSZC16	★	★			★	*IS26-orf1-orf2-orf3-fosA3-IS26*		*IS1006-virD2-floR-lysR-rcr2-glmM-sul2-IS26*	*intI1-dfrA32-aadA2-ISPpu12*			
ICE*Pmi*ChnSCDJC2	★	★			★			*IS1006-virD2-floR-lysR-rcr2-glmM-sul2-IS26*	*intI1-dfrA32-aadA2-ISPpu12*			
ICE*Pmi*ChnHBRJC2	*★*	★	★		★	*IS26-orf1-orf2-orf3-fosA3-IS26*	*ISEc59-hph-aacC4-IS26*	*IS1006-virD2-floR-lysR-rcr2-glmM-sul2-IS26*	*intI1-dfrA32-aadA2-ISPpu12*			

^*a*^ ★ represents exactly the same as the first line.

The main structure of the HS4 region, which is an important part of the entire SXT/R391 ICE, is the gene cassette composed of *IS26*, *ISPpu12*, *Tn*, and other insertion sequences, transposons, and ARGs. This indicates the crucial role of *IS26*, *ISPpu12*, *Tn*, and other insertion sequences and transposons in the accumulation of multiple ARGs and the rearrangement of regions of multidrug resistance. To date, the simultaneous presence of these elements in an ICE has not been reported, making ICE*Pmi*ChnSC1111 a unique ICE as it carries the largest number of ARGs in the SXT/R391 ICE family.

### Phylogenetic relationship of the novel multidrug-resistant SXT/R391 ICE

**Fig 3:**
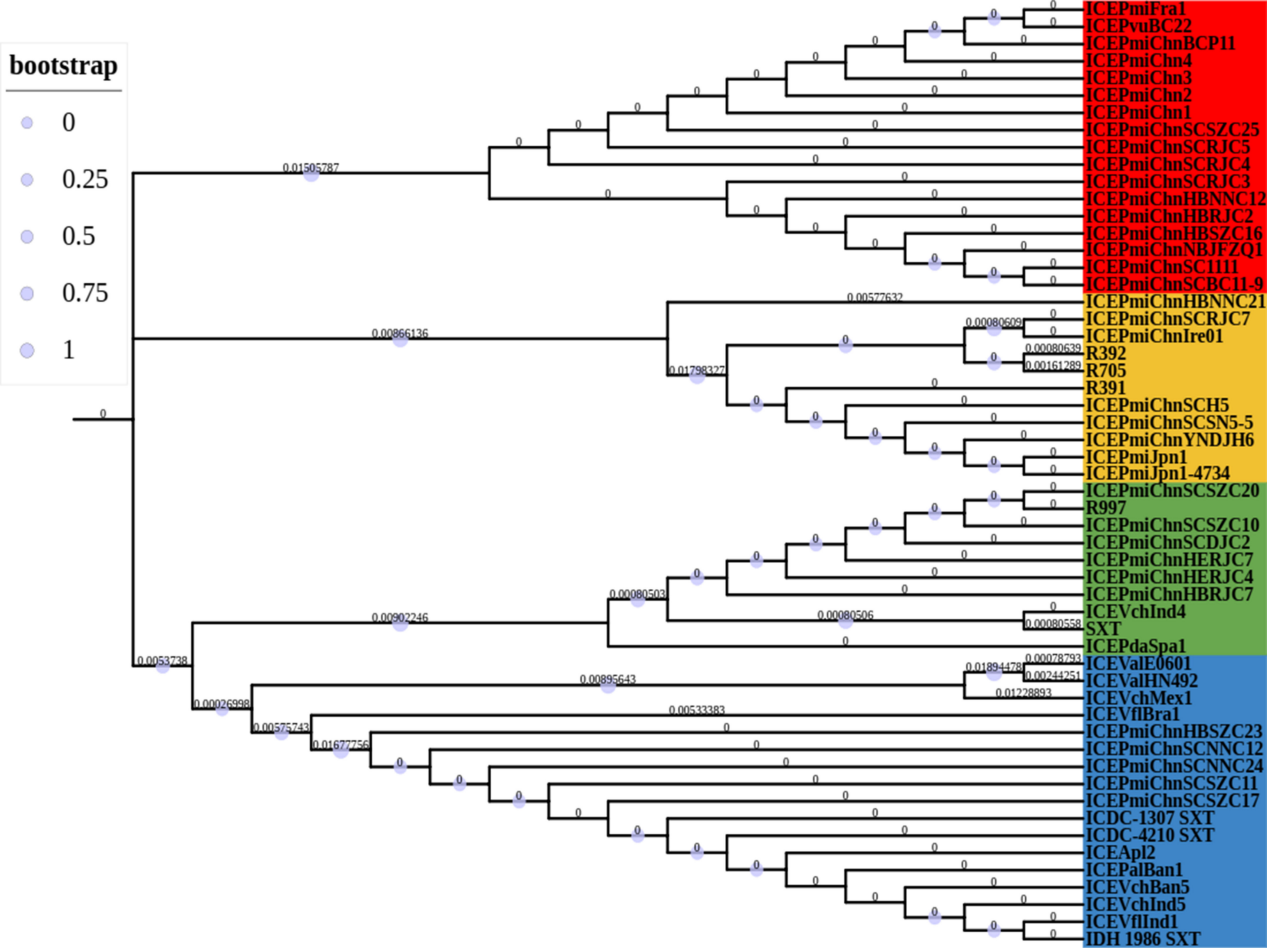
MCL phylogenetic tree based on the int genes of 55 SXT/R391 ICEs. Bootstrap analysis was calculated from 10^3^ replicates. Bar, 0.05 substitutions per nucleotide position.

**Fig 4:**
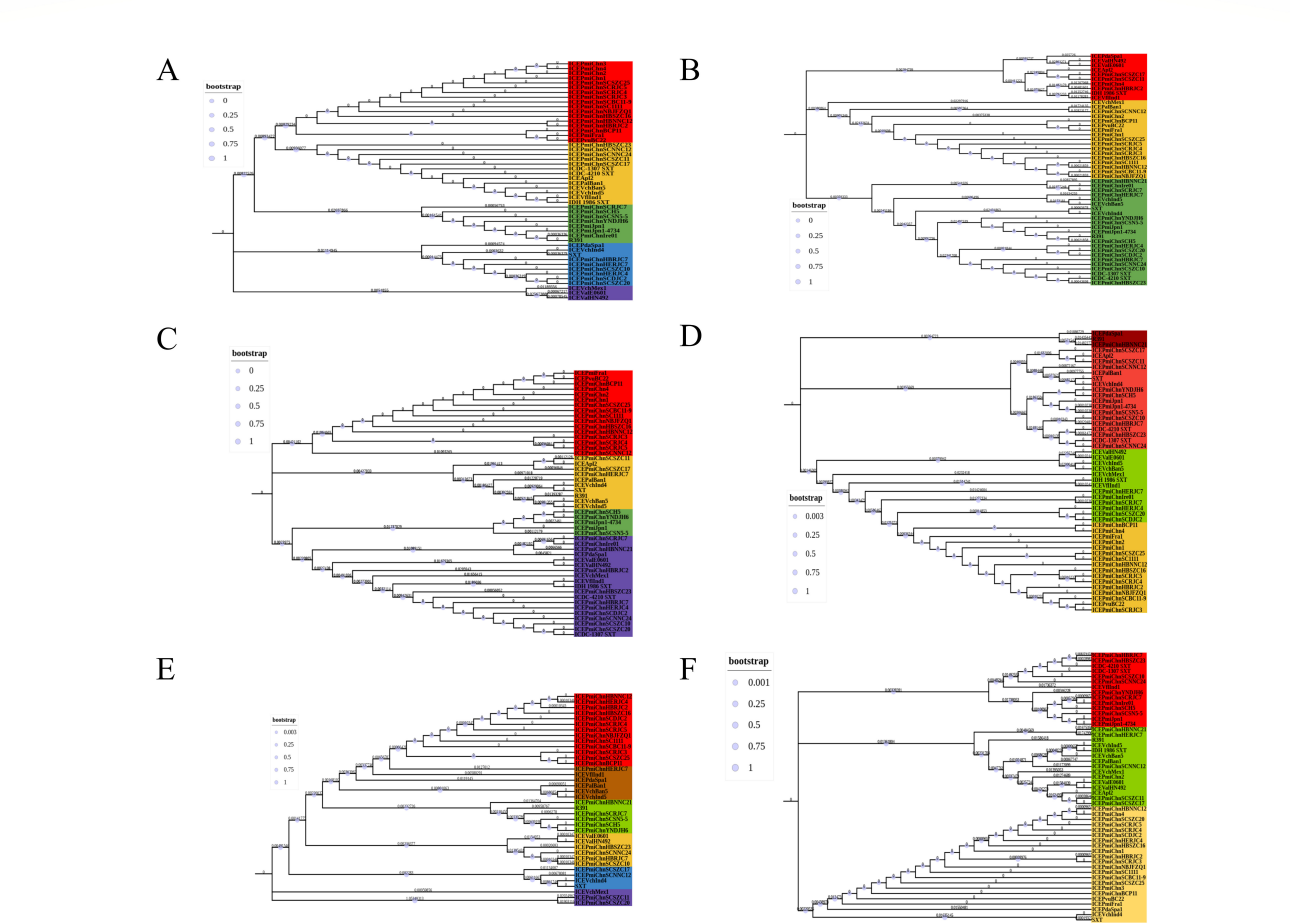
(**A**) MCL phylogenetic tree based on the VR2–VR3 region. The MCL phylogenetic tree was constructed based on the VR2–VR3 region segments of 50 SXT/R391 ICEs, resulting in the identification of four major branches. (**B**) MCL phylogenetic tree based on the HS5–HS1 region. The MCL phylogenetic tree analysis was performed on the HS5–HS1 regions of 50 SXT/R391 ICEs, and three major branches were identified. (**C**) MCL phylogenetic tree constructed based on the HS1–2 region. The MCL phylogenetic tree analysis constructed for the HS1–HS2 region of the 50 SXT/R391 ICEs revealed the formation of three major branches. (**D**) MCL phylogenetic tree based on the HS2–HS4 region. The MCL phylogenetic tree analysis was conducted on the HS2-HS4 region segments of 50 SXT/R391 ICEs, which revealed the formation of four major branches. (**E**) MCL phylogenetic tree based on the HS4–HS3 region. The MCL phylogenetic tree was constructed for the HS4–HS3 region fragments of 38 SXT/R391 ICEs, revealing a total of six major branches. (**F**) MCL phylogenetic tree based on the HS3*-attR* region. Based on the MCL phylogenetic tree of the HS3-*attR* region fragments of 51 SXT/R391 ICEs, it was found that a total of three major branches were formed. Bootstrap analysis was calculated from 10^3^ replicates. Bar, 0.05 substitutions per nucleotide position.

The maximum composite likelihood (MCL) phylogenetic tree analysis was performed on the conserved gene *int* of 55 SXT/R391 ICEs, revealing the formation of four major branches as shown in Fig. 3. The multiple sequence alignments were performed on six conserved structural regions, including hotspot regions and variable regions, of SXT/R391 ICEs to investigate their evolutionary relationships. The MCL phylogenetic tree was constructed, and the results are presented in Fig. 4. As for ICEVflBra1, R392, R705, R997, and SXT, their sequences for the conserved gene *int* were the only ones available in the NCBI database. Therefore, these five SXT/R391 ICEs were not included in the system evolutionary trees for the other conserved structural regions.

The clustering of the VR2–VR3 region of the 50 SXT/R391 ICEs is completely consistent with the clustering of the integrase gene *int*, indicating that there has been little or no evolution or that it has evolved together with *int* (Fig. 4A). The clustering of the VR2–VR3 region and the integrase gene *int* of the 50 SXT/R391 ICEs was inconsistent with that of the HS5–HS1 region, indicating that the HS5–HS1 region may have undergone more obvious evolution. (Fig. 4B). The clustering of the HS1–HS2 region and the integrase gene *int* of the 50 SXT/R391 ICEs is inconsistent, indicating that the HS5–HS1 region may have undergone more obvious evolution. (Fig. 4C). The clustering of the HS2–HS4 region of the 50 SXT/R391 ICEs and the clustering of the integrase gene *int* were inconsistent, suggesting that the HS2–HS4 region may have undergone significant evolution (Fig. 4D). The clustering of the HS4–HS3 region of 50 SXT/R391 ICEs is inconsistent with the clustering of the integrase gene *int*, indicating that the HS5–HS1 region may have undergone significant evolution (Fig. 4E). The clustering of the HS3-*attR* regions of the 51 SXT/R391 ICEs and the clustering of the integrase gene *int* are inconsistent, indicating that the HS5–HS1 region may have undergone significant evolution (Fig. 4F).

**Fig 5:**
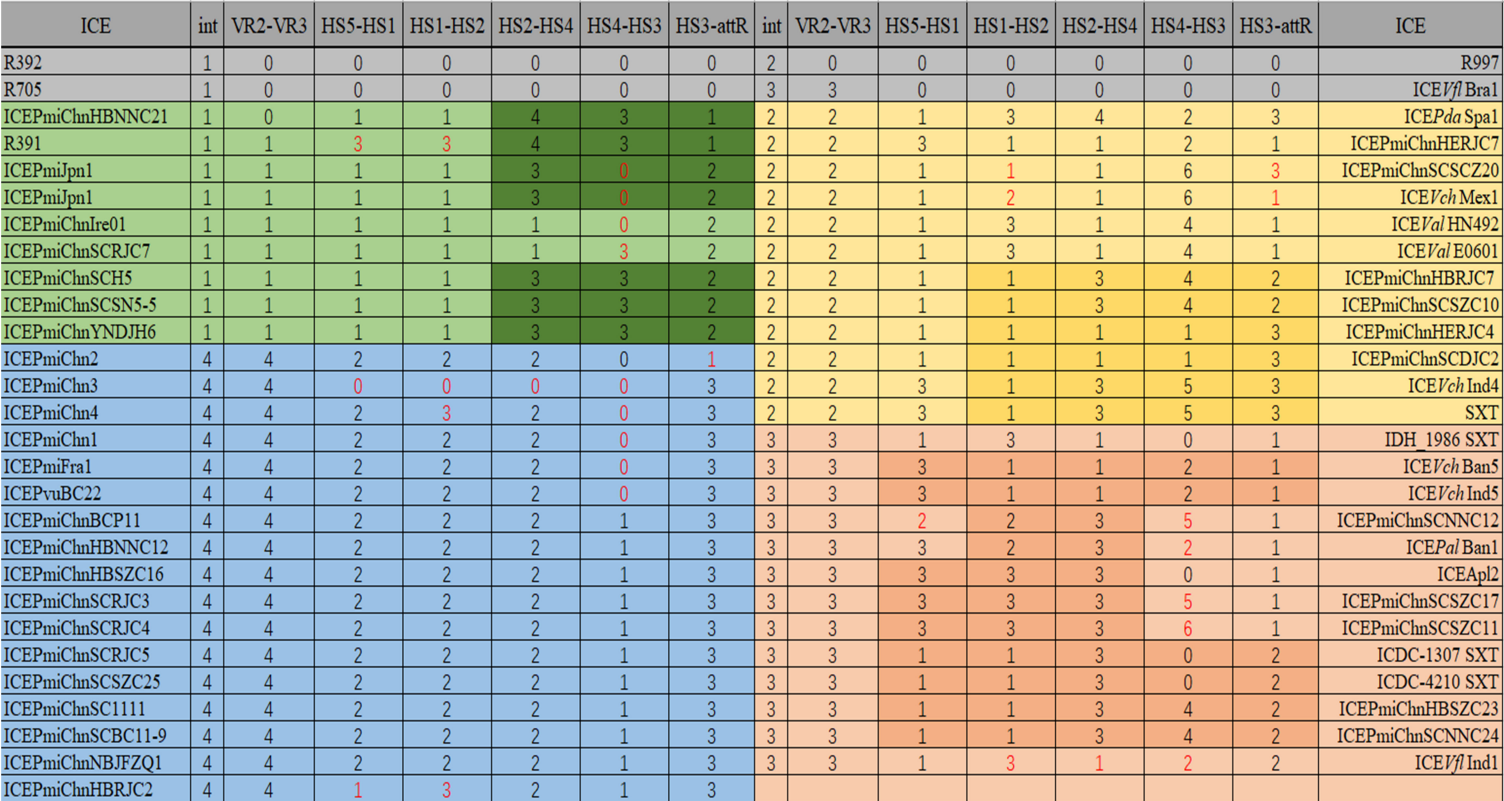
Summary of MCL phylogenetic tree of *int* gene, VR2-VR3 region, HS5-HS1 region, HS1-HS2 region, HS2-HS4 region, HS4-HS3 region, and HS3-*attR* region based on 55 SXT/R391 ICEs. Numbers 1–6 indicate phylogenetic relationships, and the same number represents a relatively close genetic relationship between the conserved genes.

The *int* gene and the HS3-*attR* region, which are located at the front and end of a single SXT/R391 ICE, respectively, and have a relatively high level of conservation, were used as the main basis for classification (Fig. 5). The summary results indicated that the evolutionary relationships of the VR2–VR3 and HS3-*attR* regions were almost consistent with that of the *int* gene, except for ICE*Pmi*ChnHBNNC21, which showed a different pattern due to the absence of the VR2–VR3 region. Although the HS3-*attR* region should theoretically maintain a relatively consistent evolutionary relationship with the *int* gene, it was found that, in addition to the two different types represented by R391 (1; *int* gene), IDH_1986 SXT (3; *int* gene), and ICE*Pmi*Chn1 (4; *int* gene), the SXT type (2) clearly showed a richer evolutionary type in the HS3-*attR* region.

### Genetic stability of SXT/R391 ICEs in donor bacteria and transconjugant

**Fig 6:**
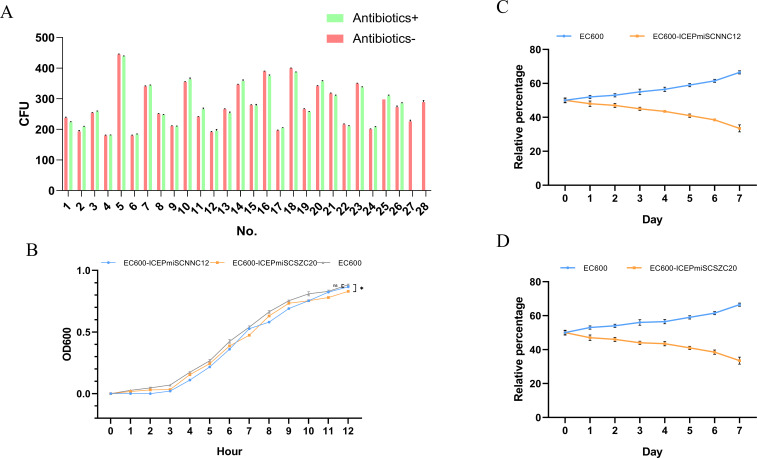
(**A**) CFU of *P. mirabilis* and transconjugant strains after 40 passages. Antibiotics: corresponding to drug resistance genes (cephalosporins or florfenicol), No.1–26 represent *P. mirabilis* strain 1–26, No. 27 represent EC600-ICEPmiChanSCNNC12, and No. 28 represent EC600-ICEPmiChnSCSZC20. (**B**) The growth curves of ICEs transconjugant and EC600. EC600-ICEPmiChnSCNNC12 represents EC600 transconjugant of ICEPmiChnSCNNC12, and EC600-ICEPmiChnSCSZC20 represents EC600 transconjugant of ICEPmiChnSCSZC20. (**C**) The competition growth curves of ICEPmiChnSCNNC12 transconjugant and EC600. (**D**) The competition growth curves of ICEPmiChnSCSZC20 transconjugant and EC600. Three parallel biological replicates experiments were performed, *, *P* < 0.05.

After 40 passages, no difference in drug resistance phenotype was observed in the *P. mirabilis* strains containing SXT/R391 ICEs compared to before, indicating that the drug resistance was maintained. The drug resistance phenotypes of EC600-ICE*Pmi*ChnSCNNC12 and EC600-ICE*Pmi*ChnSCSZC20 were consistent with the original recipient strain EC600, which was sensitive to cephalosporins (or florfenicol), indicating that the SXT/R391 ICEs in the derivatives were lost. Figure 6A shows the count results on both resistance and non-resistance SS plates for strains containing SXT/R391 ICEs. There was no significant difference with or without antibiotics, indicating that no loss of SXT/R391 ICEs occurred after the passage. However, EC600-ICE*Pmi*ChnSCNNC12 or EC600-ICE*Pmi*ChnSCSZC20 showed no growth on resistance plates. To further determine when the ICEs were lost, the PCR detection was performed, and the ICE was lost in EC600-ICE*Pmi*ChnSCNNC12 up to the 17th passage and in EC600-ICE*Pmi*ChnSCSZC20 up to the 32nd passage. In summary, SXT/R391 ICEs could be stably inherited in *P. mirabilis* but lost in EC600 during the passage.

### Growth curve of SXT/R391 ICEs transconjugant

The results of the *in vitro* growth curve after entering the logarithmic growth phase showed that the value of OD600 of transconjugant EC600-ICE*Pmi*ChnSCSZC20 were significantly lower than EC600, and there was no significant difference between the value of ICE*Pmi*ChnSCNNC12 and EC600, indicating that the growth ability of strains carrying ICE*Pmi*ChnSCSZC20 was hindered (Fig. 6B).

### Competitive growth between SXT/R391 ICEs transconjugant and the recipient stain

At the beginning of the mixed culture, the ratio of the two strains and EC600 was 50% each (Fig. 6C and D). However, as the measurements progressed, there were differences in the bacterial counts between EC600-ICE*Pmi*ChnSCNNC12, EC600-ICE*Pmi*ChnSCSZC20, and EC600, mainly reflected in the relative increase of EC600 and the corresponding decrease in EC600-ICE*Pmi*ChnSCNNC12 and EC600-ICE*Pmi*ChnSCSZC20, with this trend reaching a maximum at the final time point. In both experiments, the relative proportion of EC600 reached a maximum of 65%, while the relative proportion of EC600-ICE*Pmi*ChnSCNNC12 and EC600-ICE*Pmi*ChnSCSZC20 was only 35%. The final results indicate that EC600 exhibited varying degrees of competitive disadvantage after acquiring ICE*Pmi*ChnSCNNC12 and ICE*Pmi*ChnSCSZC20, suggesting the occurrence of adaptive costs.

## DISCUSSION

In recent years, the dissemination of multidrug resistance mediated by SXT/R391 ICEs has become a new research hotspot. *P. mirabilis* is one of the most important hosts of SXT/R391 ICEs (36). Recent studies have shown that SXT/R391 ICEs exhibit rich genetic diversity and multidrug resistance (41). Three novel multidrug-resistant SXT/R391 ICEs, ICE*Pmi*Chn2, ICE*Pmi*Chn3, and ICE*Pmi*Chn4, carrying various resistance genes, were discovered in *P. mirabilis* in 2017 (42). ICE*Pmi*ChnSTP3 was discovered in pig-derived *P. mirabilis* carrying 18 resistance genes, including *cfr* and *aac(6')-Ib-cr* (37). SXT/R391 ICEs in the *P. mirabilis* species have been shown to have rich genetic diversity and multidrug resistance by Li (43). In our previous studies, we identified ICE*Pmi*Chn1 and ICE*Pmi*ChnBCP11 in chicken-source *P. mirabilis* carrying 5 and 18 resistance genes, respectively, including clinically important rRNA methyltransferase genes *cfr*, *bla_CTX-M-65_*, *fosA3*, and *aac(6')-Ib-cr* (15, 27). In 2020, we first reported a novel SXT/R391 ICE, ICEPvuBC22, which co-carries the multidrug resistance genes *cfr* and *bla_NDM_*, and revealed its important role in the dissemination of *bla_NDM_* gene in Chinese farms (38). In this study, 21 acquired resistance genes were identified in SXT/R391 ICE-positive strains. ICE*Pmi*ChnSC1111, a newly discovered ICE, was found to carry 19 antimicrobial resistance genes, including clinically important rRNA methyltransferase gene *cfr*, *bla_CTX-M-65_*, *fosA3*, and *aac(6')-Ib-cr*. In addition, ICEPmiChnHBRJC2 carrying the erythromycin *erm (42)* resistance gene was discovered for the first time within an SXT/R391 ICE in *P. mirabilis*. The *erm (42)* gene encodes a ribosomal RNA methyltransferase that confers resistance to MLS-B (macrolides, lincosamides, and streptogramin B) type I. It contains a new mutation at A2058 in the 23S rRNA coding system of *Escherichia coli*, which has not been reported previously in ICEs of *P. mirabilis*. In 2021, Taiwanese researchers identified a new ICE, ICE_*erm (42)*, carrying the *erm (42)* gene in 26.4% of multidrug-resistant *Salmonella enterica* serovar Albany isolates from human cases of salmonellosis (44). Strains carrying ICE_*erm (42)* show high-level resistance to azithromycin, and this element can be horizontally transferred to distantly related *Vibrio cholerae*. Compared to ICE_*erm (42)*, the ICEs described in this study also carry the *floR* and *sul2* resistance genes, and some of them also carry the *strA* and *strB* genes. These findings suggest that mutations in ARGs have occurred in ICEs in mainland China and Taiwan in recent years, but differences in host types and the number and type of resistance genes still exist due to the complexity of the surrounding environment. The discovery of the newly identified erythromycin resistance gene *erm (42)* in ICE*Pmi*ChnHBRJC2 suggests that the SXT/R391 ICEs within the collection area have new resistance gene types and gene cassettes compared to the previously studied ICE*Pmi*ChnBCP11, indicating that antibiotics are still being used or that there is a certain amount of antibiotic residue in this area at the genomic level. Most of the 26 SXT/R391 ICEs discovered in this study have structures similar to that of the ICE*Pmi*ChnBCP11 structure and drug resistance genotype previously found in the vicinity (27), particularly the HS4 hot spot region, which is composed of two insertion sequences. It is suspected that, based on the similarity in collection location and time, the evolution of SXT/R391 ICEs within this range may have taken two directions: (i) evolving simultaneously from the structure of ICE*Pmi*Chn1 first found in this area, but due to differences in antibiotic usage in each region, ICE*Pmi*ChnBCP11 and ICE*Pmi*ChnSC1111 evolved into the most complex structures while evolution in the hot spot and variable regions was simpler in other areas; (ii) ICE*Pmi*Chn1 within this range initially evolved into the most complex ICE*Pmi*ChnBCP11 and ICE*Pmi*ChnSC1111, but due to human or other non-biological media transmission within the range and differences in antibiotic use, some drug resistance genes or gene cassettes were lost, resulting in the simpler structures of SXT/R391 ICEs found in this study. ICE*Pmi*ChnSC1111 and ICE*Pmi*ChnHBRJC2 have low coverage when compared with known ICEs, independent branches in the conservative core gene system phylogenetic tree, and rare insertion fragments in the hot spot regions, indicating that they are novel SXT/R391 ICEs, which will contribute to the understanding of ICEs.

ICEs are reservoirs of antibiotic resistance genes and have the ability to transfer multiple antibiotic resistance genes across different bacterial species. The host bacteria can quickly adapt to their environment and maintain stable genetic inheritance by carrying resistant ICEs (25, 45). The fitness cost of *P. mirabilis* carrying the multidrug-resistant SXT/R391 ICEs refers to the metabolic burden that the element imposes upon entering the host bacterium, which is closely related to the element’s transmission capability. When there is no antibiotic pressure, the fitness cost produced by the resistant genes on the element reduces the competitive advantage of the host in the population, leading to the loss of genes or elements (46). However, if the fitness cost brought to the host by the transfer of SXT/R391 ICEs is too low or non-existent, it weakens the cost of resistance gene mutations and poses more serious challenges and crises to the spread of resistance. In this study, with or without antibiotic pressure, ICEs can stably exist in *P. mirabilis* and confer multidrug resistance. We observed that ICEs were stably inherited in *P. mirabilis* but not in *E. coli*. The underlying reasons for this difference could be attributed to various factors, including genetic backgrounds, regulatory mechanisms, and selective pressures (40, 47). The exact mechanisms driving these differences worth further exploration. The ICEs in *P. mirabilis* can be transferred to EC600, making it resistant to antibiotics. The presence of ICEs generates benefits but also incurs a certain fitness cost (22). However, in our study, we did not specifically differentiate between spontaneous loss of the ICE from competitive disadvantage. Our observations of ICE loss in *E. coli* transconjugants were based on the disappearance of the ICE in cultures over time (Fig. 6). While this could suggest a potential fitness cost, we acknowledge that further experiments would be needed to definitively establish the cause. In addition, ICEs have a wide host range and can exist in a variety of bacteria, such as *Vibrio* spp., *E. coli*, and *Salmonella spp.* (45, 48). The SXT/R391-positive *P. mirabilis* strains in this study were isolated from farmed animals, farm workers, and the farming environment, showing the ability to resist multiple antibiotics and transfer resistance across species, posing a serious threat to public health.

The data from this study indicate that *P. mirabilis* is a crucial host and platform for the transfer, integration, and dissemination of SXT/R391 ICEs, as well as the site for the recombination and exchange of multiple antibiotic gene cassettes on the SXT/R391 ICEs. The presence of the SXT/R391 ICEs in *P. mirabilis* enhances bacterial genomic plasticity, promoting bacterial evolution to adapt to more complex and variable environments, and brings new challenges to the prevention and control of multidrug-resistant bacteria in livestock and poultry farming.

### Conclusion

In this study, 103 strains of *P. mirabilis* were isolated from 25 farms in China, and 26 of them were found to be positive for SXT/R391 ICEs. These strains exhibited high drug resistance rates and could efficiently transfer the ICEs to *E. coli* EC600. High-throughput sequencing and genome assembly were used to identify drug-resistant genes, genetic structures, homology relationships, and conserved sequences. The study found 21 drug-resistant genes and 19 different subtypes of SXT/R391 ICEs, including novel multidrug-resistant ICE*Pmi*ChnSC1111 and ICE*Pmi*ChnHBRJC2. The experiment also evaluated the adaptive cost of host bacteria after acquiring the SXT/R391 ICE through genetic stability, growth curve, and competitive ability assays. It was found that there was no significant difference in drug resistance phenotype between the donor and the transconjugant, but there was an adaptive cost in bacterial metabolism. The study provides a scientific basis for assessing the horizontal transfer and spread risk of drug-resistant genes in *P. mirabilis*.

## MATERIALS AND METHODS

### Isolation of *P. mirabilis* from farm samples

195 samples (breeding animals, breeders, and environment) were collected from 25 farms in Hebei, Hubei, and Sichuan provinces of China during the period from June 2018 to October 2020. And 103 *P. mirabilis* were isolated and detected by 16S rRNA sequencing, including 15 strains from breeders, 58 strains from pigs, 16 strains from chickens, and 14 strains from cattle.

The sample collected from the farm is directly cultured in brain heart infusion (BHI) medium (Land Bridge, Beijing, China) and shaken overnight at 37℃. The mixed culture is streaked onto eosin methylene blue (EMB) solid medium (Land Bridge, Beijing, China) using a sterile inoculating loop and incubated inverted overnight at 37℃. Suspicious translucent or black flat colonies are observed and, if present, are streaked onto BHI medium using a sterile inoculating loop and shaken overnight at 37℃. The obtained bacterial liquid is preserved with 99% glycerol in the same ratio. Meanwhile, single colonies of the culture are streaked onto SS medium (Land Bridge, Beijing, China) using a sterile inoculating loop and incubated inverted overnight at 37℃ to observe the characteristic migration of *P. mirabilis*. If migration occurs, the single colony is basically identified as *P. mirabilis*. To confirm more accurately, the 16S rDNA PCR is performed to confirm the identification at the genomic level.

### Detection of SXT/R391 ICEs and the circular extrachromosomal forms

PCR was performed using the DNA template and primers targeting the *int* gene (which is located very close to the 5′ end of the SXT/R391 ICE, is relatively conserved compared to other genes, and typically encodes the integrase that facilitates integration into the bacterial genome), the *attL* site (the left end region of the SXT/R391 ICE formed after specific recombination between the *attP* site on the ICE and the *attB* site located at the 5′ end of the *prfC* gene on the recipient bacterial chromosome, under the action of the integrase *int*), and the *attR* site (the right end region of the SXT/R391 ICE formed after specific recombination between the *attP* site on the ICE and the *attB* site located at the 5′ end of the *prfC* gene on the recipient bacterial chromosome, under the action of the integrase *int*). The PCR products were then analyzed by agarose gel electrophoresis, and the amplified DNA fragments close to the target band were sequenced using Sanger sequencing technology to determine whether the SXT/R391 ICE backbone sequence was present in the *P. mirabilis*, and therefore, whether the SXT/R391 ICE was present in the *P. mirabilis* (Table S1).

### Antimicrobial susceptibility tests

The antibiotic susceptibility of *P. mirabilis* was carried out by using the Kirby-Bauer method according to the Clinical and Laboratory Standards Institute (CLSI) document M02-A11 (11) and the guidelines of the European Committee on Antimicrobial Susceptibility Testing (EUCAST; http://www.eucast.org, accessed on 9 May 2019). Determine the diameter of each bacteriostatic ring according to CLSI (CLSI document M100 and M31-ST) drug sensitivity interpretation standard (Table S4).

### Genomic sequencing and analysis

*P. mirabilis* was cultured in BHI medium until the OD600 reached 0.7, and genomic DNA was extracted by using the TIANamp Bacteria DNA Kit (TIANGEN, Beijing, China). The quality and purity of the extracted genomic DNA were evaluated using 0.1% agarose gel electrophoresis. NanoDrop2000 was used to measure the concentration and the values of OD260/280 and OD260/230 of DNA samples. The Qubit method was used to verify the concentration of DNA. These three methods were used in combination to ensure that the concentration and purity of the genomic DNA met the requirements for Illumina Miseq and Nanopore third-generation high-throughput sequencing. One portion of the total DNA was sent to a sequencing company (Sangon Biotech, Shanghai, China) for high-throughput sequencing using the Illumina Miseq X150 platform, while the other portion was subjected to Nanopore third-generation sequencing in our laboratory.

In order to investigate whether there is any duplicate cloning among 26 SXT/R391 ICE-positive *P. mirabilis* isolates, single nucleotide polymorphism (SNP) typing was used to evaluate the spliced data in order to eliminate interference in subsequent experiments. The Center for Genomic Epidemiology’s CSIPhylogeny1.4 tool (https://cge.food.dtu.dk/services/CSIPhylogeny/) was used to detect and type the 26 SXT/R391 ICE-positive *P. mirabilis* isolates, with the data from BB2000 (No. CP004022) used as reference. A mutation type proportion of 80% or higher at a certain site is considered a fixed mutation, and highly repetitive regions, GC-enriched sequences, and drug-resistant gene SNPs are excluded. The SNP must be measured in at least 10 reads without positive or negative strand bias. The returned results were used to construct an MCL tree, and the full genome phylogenetic tree of the 26 SXT/R391 ICE-positive *P. mirabilis* isolates was constructed using MCL method in iTOL (https://itol.embl.de/).

### Design of bridge PCR primers

The concentration of extracted DNA was 100–140 μg/μL, the value of OD260/OD280 was 1.7–1.8, the sequencing depth was 227×–275×, and the whole genome sequence was spliced into 85–160 Scaffolds. In the isolates, PmHBNNC21 and PmSCRJC7, the complete ICEs were found on a single Scaffold. However, in PmHBRJC7, PmHBSZC16, PmHBSZC23, PmHERJC4, PmHERJC7, PmSCNNC12, PmSCNNC24, PmSCRJC4, PmSCRJC5, PmSCRJC7, PmSCSZC10, PmSCSZC11, PmSCSZC17, PmSCSZC20, PmSCSZC25, PmSCBC11-9, PmSCSN5-5, PmYNDJH6, and PmNBJFZQ1, the ICE fragments were distributed within 10 Scaffolds. The structures of the ICE fragments were very similar to ICE*Pmi*Jpn1, ICDC-1307 SXT, IDH_ 1986 SXT, ICEApl2, ICDC-4210 SXT, and ICE*Pmi*Fra1. The gap region was amplified using primers designed based on the above-referenced ICE alignment genome sequence. The splicing between two adjacent Scaffolds was completed by bridge PCR and first-generation Sanger sequencing, and the shared sequence obtained was used to obtain the complete sequence of ICEs. The primer sequences are shown in Table S5. In PmHBNNC12, PmHBRJC2, PmSCDJC2, PmSCRJC3, and PmSC1111, the ICE fragments were distributed on more than 10 Scaffolds, and their structures were more complex. The third generation of nanopore sequencing was used to obtain the complete structure. The complete genome was assembled and spliced by combining the Illumina and Nanopore data.

### Genetic structure analysis of novel multidrug-resistant SXT/R391 ICEs

The assembled data of *P. mirabilis* were analyzed for acquired ARGs using database tools. The ResFinder tool provided by the Center for Genomic Epidemiology (http://www.genomicepidemiology.org/services/) was used to determine the types and locations of acquired resistance genes in *P. mirabilis*. The default similarity threshold was set at 90%, and the minimum alignment length was set at 60%. Quality filtering of Illumina reads was performed, and adapters were removed using trimomatic v.0.392 (49). For the passing reads, a minimum quality threshold of 20 was set. SPAdes v.3.11 was used for *de novo* assembly (50). For the Oxford Nanopore Technologies (ONT) reads, quality filtering was performed using guppy 2.1.3 (51), and the Unicycler software (52) was used to obtain hybrid assemblies. Annotation was performed using the NCBI Prokaryotic Genome Annotation Pipeline (53). The completed *P. mirabilis* genome was roughly annotated using the RAST software (54) to obtain basic gene information. The Scaffolds containing the SXT/R391 ICE fragments were identified using the BLAST tool provided by NCBI (http://ncbi.nlm.nih.gov/BLAST). All Scaffolds that potentially contained SXT/R391ICE fragments were arranged according to the reference sequence of the SXT/R391 ICE, and PCR primers were designed at the two ends of each gap. Only the 5′ end of the synthesized primer pair and the 3' end of the next primer pair were retained, and they were combined to form a new primer pair that theoretically covered the entire gap, the end of the known sequence in the front, and the beginning of the known sequence in the back. The PCR products were subjected to Sanger sequencing, and the results were aligned with the known sequences from the front and the back to obtain the gene sequence in the gap. This process was repeated for all Scaffolds that contained SXT/R391 ICE fragments to obtain the complete gene sequence of a single SXT/R391 ICE.

In theory, a single-end Sanger sequencing reaction can only obtain gene sequences of about 1,500 bp, so if the gene sequence in the gap missing region between two adjacent scaffolds exceeds this range, it is necessary to design multiple pairs of bridging PCR primers or use nanopore third-generation whole-genome sequencing methods to solve the problem. In this study, ICE fragments of PmHBNNC12, PmHBRJC2, PmSCDJC2, PmSCRJC3, and PmSC1111 are distributed on more than 10 scaffolds, and the gap missing region in the middle greatly exceeds the range of a single-end Sanger sequencing reaction and is more complex in structure. In order to obtain a complete sequence structure, nanopore third-generation sequencing was used alone, and the Illumina and Nanopore data were combined for assembly and splicing into a complete genome. After obtaining the complete gene sequences of all individual SXT/R391 ICEs, they were analyzed in more detail and comprehensively annotated using the bacterial genome annotation software Sequin (https://www.ncbi.nlm.nih.gov/Sequin/) in combination with the RAST results obtained at the beginning. The annotated results were submitted to the Genebank database using the Bankit tool provided by NCBI to obtain a Genebank accession number. In the analysis process, the previous ICE sequences in NCBI were used as references, and the Easyfig software (version 2.2.2) (55) was used to compare the SXT/R391 ICEs discovered in this study with the most typical or similar ICEs in the database and then draw a genetic structure map for a more intuitive and clear analysis of their genetic structure and function.

### Analysis of the phylogenetic relationship of novel multidrug-resistant SXT/R391 ICEs

To investigate the phylogenetic relationships among the 26 SXT/R391 ICEs identified in this study (number 30–55 in Table S3) and those previously reported, as well as the evolutionary relationships between different backbone regions of the 26 ICEs, we downloaded the gbk format sequence files of 29 SXT/R391 ICEs (number 1–29 in Table S3) from the Genebank database. We then used MEGAX software (version 7.0.5) based on the Tamura-Nei model and MCL method to perform multiple sequence alignment of all 55 backbone regions of SXT/R391 ICEs, including the integrase gene *int*, the gene sequence region between VR2 and VR3, the gene sequence region between HS5 and HS1, the gene sequence region between HS1 and HS2, the gene sequence region between HS2 and HS4, the gene sequence region between HS4 and HS3, and the gene sequence region between HS3 and *attR*. We then constructed an MCL phylogenetic tree to analyze the evolutionary relationships between different backbone regions. Since the HS3-*attR* region of R391 contains a *mer* operon gene cluster that is absent in other SXT/R391 ICEs, we decided to exclude this region from the alignment to ensure relative consistency of the results.

### Conjugative transfer of SXT/R391 ICEs

Measurement of conjugative transfer capability was performed using filter mating assay. The donor strains (26 *P. mirabilis* carrying SXT/R391 ICEs) and the rifampin-resistant strain *E. coli* EC600 (Huayueyang, Beijing) were cultured to the logarithmic phase at 37°C. 50 uL of each strain was taken and thoroughly mixed by gentle pipetting onto a sterile 0.22 µm pore-size filter membrane placed on an MH agar plate, followed by overnight incubation at 37°C in a static incubator. The mixed culture was then washed off with a sterile BHI medium, and serial dilutions (original, 10×, 10^2^×, and 10^5^× dilutions) were made. Aliquots of 100 µL from each dilution were evenly spread onto EMB agar plates containing 300 µg/mL rifampicin and either 4 µg/mL cefotaxime or 8 µg/mL florfenicol, and onto rifampicin-containing plates for 10^5^× dilution. The plates were incubated overnight, and the number of colonies on each plate was counted to determine the bacterial count, as well as the transfer frequency of the donor to recipient cells. Five suspected transconjugants from each double-antibiotic-containing plate were subjected to species identification and PCR detection of the integrase gene *int* within ICEs, as mentioned earlier. The recipient strain EC600 and transconjugants were subjected to antibiotic susceptibility testing using the K-B paper disk method to confirm successful conjugation transfer, as mentioned earlier.

### Genetic stability testing of SXT/R391 ICEs

To investigate the genetic stability of SXT/R391 ICEs in *P. mirabilis* and its transconjugant (selecting ICE*Pmi*ChnSCNNC12 and ICE*Pmi*ChnSCSZC20 as representatives), *P. mirabilis* and EC600 transconjugant carrying SXT/R391 ICEs were consistently cultured for 40 passages in the absence of antibiotic selection pressure, with each passage being 16 hours and lasting approximately 30 days. The final bacterial liquid was subjected to antibiotic susceptibility testing to observe whether the drug-resistant phenotype of the strains had changed. The final bacterial liquid was diluted 10^4^× and cultured on both resistant and non-resistant SS plates, with three parallel sets for each, to count the number of colonies and perform PCR detection of conserved genes of SXT/R391 ICEs (integrase gene *int*, *attL* site region, and *attR* region) to determine whether loss of SXT/R391 ICEs had occurred during passage culture. At the same time, it was determined whether homologous recombination had occurred between multiple antibiotic resistance regions during passage culture.

### Determination of the growth curve of the EC600 carrying SXT/R391 ICEs

EC600-ICE*Pmi*ChnSCNNC12, EC600-ICE*Pmi*ChnSCSZC20 ICEs, and control group EC600 were co-cultured on EMB agar. Then, the culture was transferred to BHI broth and incubated at 37°C for 12 hours, and the OD600 was measured and calibrated using BHI broth (OD600 = 0.6). Next, 1 mL of the cultured broth was taken using a sterile pipette and added to 100 mL of BHI broth, followed by incubation in a constant temperature shaker at 37°C and 180 rpm. The mixing time was set to 0, and 200 µL of the culture was taken every hour and transferred to a 96-well plate for OD600 measurement.

### Competitive growth test of the EC600 carrying SXT/R391 ICEs

EC600-ICE*Pmi*ChnSCNNC12, EC600-ICE*Pmi*ChnSCSZC20, and control group EC600 were mixed in the same proportion at the same dilution, and 100 µL was inoculated into 10 mL of BHI medium. The time was set as 0, and the mixture was incubated at 37°C and 180 rpm with three parallel cultures for six cycles, i.e., 6 days. At each cycle, 100 µL of the culture was transferred into 10 mL of fresh BHI medium and incubated at 37°C and 180 rpm. After seven cycles, the bacterial liquid was diluted with 0.9% saline solution at dilutions of 10^2^×, 10^5^×, 10^6^×, and 10^7^×. To count transconjugants, EMB agar medium containing 8 µg/mL florfenicol was used. Simultaneously, EMB agar medium without antibiotics was used to count the total bacterial population, including both transconjugants and the control strain.

### Statistical analysis

All statistical analyses were performed using GraphPad Prism 8 (GraphPad Software). Statistical analyses were performed using data from three biological replicates, using Student’s *t* tests. Results represent the mean ± SD. *P* > 0.05, labeled ns, *P* < 0.05, labeled *.

## Data Availability

The accession numbers of all genomes sequenced in this study or downloaded from NCBI are shown in Table 1, as well as Tables S2 and S3.

## SUPPLEMENTAL MATERIAL

Fig. S1 spectrum.01209-23-s0001.pdf The detection of the SXT/R391 ICEs gene in 26 *P. mirabilis* isolates.

Table S1 spectrum.01209-23-s0002.docx Primers used to detect the SXT/R391 ICE.

Table S2 spectrum.01209-23-s0003.docx Detailed information of *P. mirabilis* strains from other regions in China.

Table S3 spectrum.01209-23-s0004.docx GenBank no. of SXT/R391 ICEs in MCL system analysis.

Table S4 spectrum.01209-23-s0005.docx Criterion of *P.* mirabilis for drug susceptibility test.

Table S5 spectrum.01209-23-s0006.docx The bridge PCR primers of SXT/R391 ICEs.

ASM does not own the copyrights to Supplemental Material that may be linked to, or accessed through, an article. The authors have granted ASM a non-exclusive, world-wide license to publish the Supplemental Material files. Please contact the corresponding author directly for reuse.
