# Supplementary material for: Characterization and risk assessment of novel SXT/R391 integrative and conjugative elements with multidrug resistance in Proteus mirabilis isolated from China, 2018–2020
Source: Microbiol Spectr. 2024 Jan 10;12(2):e01209-23. doi: 10.1128/spectrum.01209-23 (PMC10871549; doi:10.1128/spectrum.01209-23)
Supplement: Fig. S1 — The detection of the SXT/R391 ICEs gene in 26 P. mirabilis isolates. [file spectrum.01209-23-s0001.pdf]

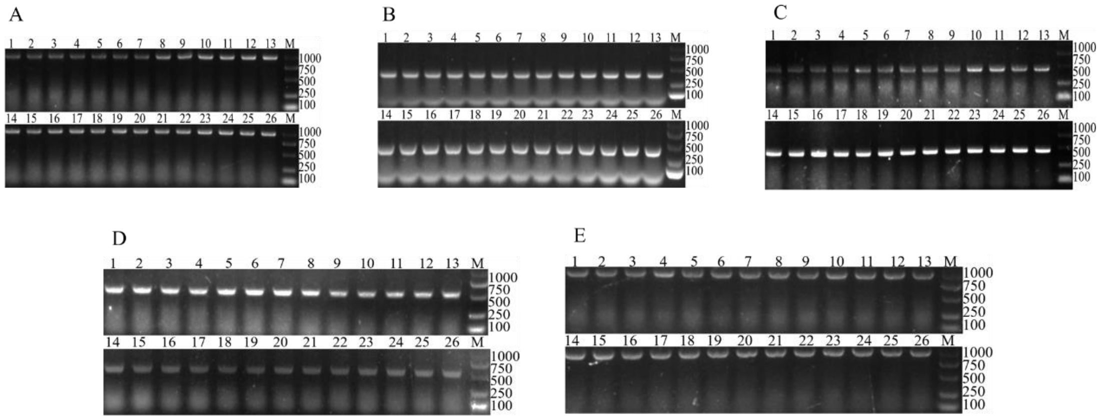

Fig. S1 The detection of the SXT/R391 ICEs gene in 26 *P. mirabilis* isolates. (A) The detection of *int* gene. (B) The detection of *attL* site. (C) The detection of *attR* site. (D) The detection of the circular extrachromosomal forms. (E) The detection of *int* gene in the transconjugants of 26 *P. mirabilis* isolates. The agarose gel electrophoresis results of PCR products showed that 26 strains of *P. mirabilis* were positive for SXT/R391, as indicated by the presence of *int* gene, *attL* site, and *attR* site. The positive bands of approximately 550bp, which represented circular extrachromosomal forms, were detected in 26 *P. mirabilis* strains. Sequencing of the PCR products confirmed a shared nucleotide identity of over 98% when compared to the target gene sequence. 1-26 as listed in Table 1. M, Marker2000.
