# Supplementary material for: Characterization and risk assessment of novel SXT/R391 integrative and conjugative elements with multidrug resistance in Proteus mirabilis isolated from China, 2018–2020
Source: Microbiol Spectr. 2024 Jan 10;12(2):e01209-23. doi: 10.1128/spectrum.01209-23 (PMC10871549; doi:10.1128/spectrum.01209-23)
Supplement: Table S1 — Primers used to detect the SXT/R391 ICE. [file spectrum.01209-23-s0002.docx]

**Table S1 Primers used to detected the SXT/R391 ICE**

| **Primer** | **Sequence** | **Target** | **Length(bp)** |
| --- | --- | --- | --- |
| 16SrDNA-F | 5’-AGAGTTTGATCMTGGCTCAG-3’ | 16SrDNA | ～1400bp |
| 16SrDNA-R | 5’-GGTTACCTTGTTACGACTT-3’ |  |  |
| *int*-F | 5’-CTGTGGCCAATCATCAACTC-3’ | *int* | 1000bp |
| *int*-R | 5’-CGACCGAGATGGGCTAAGTG-3’ |  |  |
| *attL*-F | 5’-ACAACGACAACAGAGCATTGG-3’ | *attL* | 413bp |
| *attL*-R | 5’-GTACACACTTTCCGAGGTTACG-3’ |  |  |
| *attR*-F | 5’-TGCACGTTGGATAGCTTGTCCG-3’ | *attR* | 466bp |
| *attR*-R | 5’-CCGCAATACCCTGCAATACCGA-3’ |  |  |
| circular -F | 5’-GTACACACTTTCCGAGGTTACG-3’ | circular extrachromosomal forms | 550bp |
| circular-R | 5’-CCGCAATACCCTGCAATACCGA-3’ |  |  |
