# Supplementary material for: Characterization and risk assessment of novel SXT/R391 integrative and conjugative elements with multidrug resistance in Proteus mirabilis isolated from China, 2018–2020
Source: Microbiol Spectr. 2024 Jan 10;12(2):e01209-23. doi: 10.1128/spectrum.01209-23 (PMC10871549; doi:10.1128/spectrum.01209-23)
Supplement: Table S2 — Detailed information of P. mirabilis strains from other regions in China. [file spectrum.01209-23-s0003.docx]

**Table S2. Detailed information of *P. mirabilis* strains from other region in China (Download from NCBI) used in the phylogenetic tree**

| **Accession no.** | **Strain** | **Province** | **Year** | **Source** |
| --- | --- | --- | --- | --- |
| CP026571 | BC11-24 | Sichuan | 2016 | Pig |
| CP053894 | JPM24 | Guangdong | 2017 | Chicken |
| CP053898 | YPM35 | Guangdong | 2017 | Chicken |
| CP065147 | PmBJ015-2 | Beijing | 2017 | Humans |
| CP065148 | PmBJ012-2 | Beijing | 2017 | Humans |
| CP044436 | C55 | Shandong | 2018 | Pig |
| CP066833 | RGF134-1 | Jiangsu | 2019 | Pig |
| CP047589 | SNYG35 | Sichuan | 2018 | Chicken |
| JAMKNU01 | PM2-3 | Zhejiang | 2021 | Pig |
| JAMKOC01 | PM1-4 | Zhejiang | 2021 | Pig |
| JAMKOF01 | PM1-1 | Zhejiang | 2021 | Pig |
|  |  |  |  |  |
