# Supplementary material for: Characterization and risk assessment of novel SXT/R391 integrative and conjugative elements with multidrug resistance in Proteus mirabilis isolated from China, 2018–2020
Source: Microbiol Spectr. 2024 Jan 10;12(2):e01209-23. doi: 10.1128/spectrum.01209-23 (PMC10871549; doi:10.1128/spectrum.01209-23)
Supplement: Table S3 — GenBank no. of SXT/R391 ICEs in MCL system analysis. [file spectrum.01209-23-s0004.docx]

**Table S3 GenBank no. of SXT/R391 ICEs in MCL system analysis**

| **Number** | **SXT/R391 ICE** | **Host** | **GenBank NO.** | **Number** | **SXT/R391 ICE** | **Host** | **GenBank NO.** |
| --- | --- | --- | --- | --- | --- | --- | --- |
| 1 | ICE*Val*HN492 | *Vibrio alginolyticus* | KT072769 | 29 | ICE*Pmi*Jpn1 | *Proteus mirabilis* | KT894734 |
| 2 | ICE*Val*E0601 | *Vibrio alginolyticus* | KT072768 | 30 | ICE*Pmi*ChnHBNNC12 | *Proteus mirabilis* | MZ277865 |
| 3 | ICE*Vch*Mex1 | *Vibrio cholerae* | GQ463143 | 31 | ICE*Pmi*ChnHBNNC21 | *Proteus mirabilis* | MW856286 |
| 4 | ICE*Pda*Spa1 | *Photorhabdus luminescens* | AJ870986 | 32 | ICE*Pmi*ChnHBRJC2 | *Proteus mirabilis* | MW978700 |
| 5 | R997 | *Proteus mirabilis* | AJ634266 | 33 | ICE*Pmi*ChnHBRJC7 | *Proteus mirabilis* | MZ221989 |
| 6 | ICE*Vch*Ind4 | *Vibrio cholerae* | GQ463141 | 34 | ICE*Pmi*ChnHBSZC16 | *Proteus mirabilis* | MZ277866 |
| 7 | SXT | *Vibrio cholerae* | AY055428 | 35 | ICE*Pmi*ChnHBSZC23 | *Proteus mirabilis* | MZ052215 |
| 8 | ICE*Vfl*Bra1 | *Vibrio fluvialis* | JQ180502 | 36 | ICE*Pmi*ChnHERJC4 | *Proteus mirabilis* | MZ221994 |
| 9 | ICE*Pal*Ban1 | *Providencia Ewing* | GQ463139 | 37 | ICE*Pmi*ChnHERJC7 | *Proteus mirabilis* | MZ005587 |
| 10 | ICE*Vch*Ban5 | *Vibrio cholerae* | GQ463140 | 38 | ICE*Pmi*ChnSCDJC2 | *Proteus mirabilis* | MZ277867 |
| 11 | ICE*Vch*Ind5 | *Vibrio cholerae* | GQ463142 | 39 | ICE*Pmi*ChnSCNNC12 | *Proteus mirabilis* | MZ005586 |
| 12 | ICE*Vfl*Ind1 | *Vibrio fluvialis* | KM213605 | 40 | ICE*Pmi*ChnSCNNC24 | *Proteus mirabilis* | MZ005588 |
| 13 | R391 | *Providencia rettgeri* | AY090559 | 41 | ICE*Pmi*ChnSCRJC3 | *Proteus mirabilis* | MW821905 |
| 14 | R392 | *Providencia rettgeri* | AJ634264 | 42 | ICE*Pmi*ChnSCRJC4 | *Proteus mirabilis* | MZ221992 |
| 15 | R705 | *Proteus vulgaris* | AJ634265 | 43 | ICE*Pmi*ChnSCRJC5 | *Proteus mirabilis* | MZ221993 |
| 16 | ICE*Pmi*ChnBCP11 | *Proteus mirabilis* | MG773277 | 44 | ICE*Pmi*ChnSCRJC7 | *Proteus mirabilis* | MZ052212 |
| 17 | ICE*Pmi*Chn1 | *Proteus mirabilis* | KT962845 | 45 | ICE*Pmi*ChnSCSZC10 | *Proteus mirabilis* | MZ052217 |
| 18 | ICE*Pmi*Fra1 | *Proteus mirabilis* | MF490434 | 46 | ICE*Pmi*ChnSCSZC11 | *Proteus mirabilis* | MW978701 |
| 19 | ICEPmiChn2 | *Proteus mirabilis* | KY437726 | 47 | ICE*Pmi*ChnSCSZC17 | *Proteus mirabilis* | MZ052214 |
| 20 | ICE*Pmi*Jpn1 | *Proteus mirabilis* | KY437729 | 48 | ICE*Pmi*ChnSCSCZ20 | *Proteus mirabilis* | MZ221996 |
| 21 | ICE*Pmi*Chn3 | *Proteus mirabilis* | KY437727 | 49 | ICE*Pmi*ChnSCSZC25 | *Proteus mirabilis* | MZ052216 |
| 22 | ICE*Pmi*Chn4 | *Proteus mirabilis* | KY437728 | 50 | ICE*Pmi*ChnSCH5 | *Proteus mirabilis* | MZ052211 |
| 23 | ICDC-1307 SXT | *Vibrio cholerae* | KJ817376 | 51 | ICE*Pmi*ChnSC1111 | *Proteus mirabilis* | MW978699 |
| 24 | ICE*Pvu*BC22 | *Proteus vulgaris* | MH160822 | 52 | ICE*Pmi*ChnSCBC11-9 | *Proteus mirabilis* | MZ221991 |
| 25 | IDH_1986 SXT | *Vibrio cholerae* | MK165649 | 53 | ICE*Pmi*ChnSCSN5-5 | *Proteus mirabilis* | MZ052213 |
| 26 | ICE*Pmi*ChnIre01 | *Proteus mirabilis* | MN520463 | 54 | ICE*Pmi*ChnYNDJH6 | *Proteus mirabilis* | MZ221995 |
| 27 | ICE*Apl*2 | *Actinobacillus Pleuropneumoniae* | MF187965 | 55 | ICE*Pmi*ChnNBJFZQ1 | *Proteus mirabilis* | MZ221990 |
| 28 | ICDC-4210 SXT | *Vibrio cholerae* | KT151662 |  |  |  |  |
