# Supplementary material for: Characterization and risk assessment of novel SXT/R391 integrative and conjugative elements with multidrug resistance in Proteus mirabilis isolated from China, 2018–2020
Source: Microbiol Spectr. 2024 Jan 10;12(2):e01209-23. doi: 10.1128/spectrum.01209-23 (PMC10871549; doi:10.1128/spectrum.01209-23)
Supplement: Table S4 — Criterion of P. mirabilis for drug susceptibility test. [file spectrum.01209-23-s0005.docx]

**Table S4 Criterion of *P. mirabilis* for drug susceptibility test**

| **Antibiotics** | **Content（μg/per slice）** | **Criterion（mm）** | | |
| --- | --- | --- | --- | --- |
|  |  | **R（≦）** | **I** | **S（≥）** |
| FFC | 30 | 14 | 14-18 | 19 |
| CAZ | 30 | 17 | 18-20 | 21 |
| FOX | 30 | 14 | 15-17 | 18 |
| NAL | 30 | 13 | 14-18 | 19 |
| CTX | 30 | 22 | 23-25 | 26 |
| AMC | 30 | 13 | 14-17 | 18 |
| ATM | 30 | 17 | 18-20 | 21 |
| AMP | 10 | 13 | 14-16 | 17 |
| SXT | 25 | 10 | 11-15 | 16 |
| NOR | 10 | 12 | 13-15 | 16 |
| CIP | 5 | 15 | 16-20 | 21 |
| GEN | 10 | 12 | 13-14 | 15 |
| AMK | 30 | 14 | 15-16 | 17 |
| SPT | 100 | 10 | 11-13 | 14 |
| STR | 10 | 11 | 12-14 | 15 |
| IPM | 10 | 19 | 20-22 | 23 |

FFC, Florfenicol; CAZ, Ceftazidime; FOX, Cefoxitin; NAL, Nalidixic acid; CTX, Cefotaxime; AMC, Amoxycillin/clavulanic acid; ATM, Aztreonam; AMP, Ampicillin;

SXT, Sulphamethoxazole/trimethoprim; NOR, Norfloxacin; CIP, Ciprofloxacin; GEN, Gentamicin; AMK, Amikacin; SPT, Spectinomycin; STR, Streptomycin; IPM, Imipenem;
