# Supplementary material for: Characterization and risk assessment of novel SXT/R391 integrative and conjugative elements with multidrug resistance in Proteus mirabilis isolated from China, 2018–2020
Source: Microbiol Spectr. 2024 Jan 10;12(2):e01209-23. doi: 10.1128/spectrum.01209-23 (PMC10871549; doi:10.1128/spectrum.01209-23)
Supplement: Table S5 — The bridge PCR primers of SXT/R391 ICEs. [file spectrum.01209-23-s0006.docx]

**Table S5 The bridge PCR primers of SXT/R391 ICEs**

| **Strain** | **Primer** | **Sequence** | **Lenth（bp）** |
| --- | --- | --- | --- |
| ICE*Pmi*ChnHERJC4 | ICEApl2.09-tnp-F | TGCGTCATTACGTCCAGCAA | 1209 |
|  | ICEApl2.09-tnp-R | TGTTGTGCAAAGGTGTTGGC | 1209 |
|  | ICEApl2.16-tnpA-F | CTGGCAGGATACCGCTCATT | 906 |
|  | ICEApl2.16-tnpA-R | CAGGTGCGACAGACGTCATA | 906 |
|  | ICEApl2.11-12-F | CTGCCGACCAAAAGCGCTAC | 1600 |
|  | ICEApl2.11-12-R | GGCGTTCCTCTGGGCTAAAC | 1600 |
|  | ICEApl2.12-13-F | ACCAAGACGCTGGTCAACAA | 1578 |
|  | ICEApl2.12-13-R | TAGGGTCTTCAATGCAGGCG | 1578 |
|  | sul2-ICEApl2.21-F | GACAGAAGCACCGGCAAATC | 1537 |
|  | sul2-ICEApl2.21-R | ATCAAAGAGGTAACGCGGCT | 1537 |
|  | ICEApl2.21-F | GAACGATGCGGAATTGGCAG | 1509 |
|  | ICEApl2.21-R | CAATGTGGTGCCAGTGAATCT | 1509 |
|  | ICEApl2.21-22-F | ATGGCGTTATCGATGGGCTT | 1533 |
|  | ICEApl2.21-22-R | CGAAACATGCGCCGTAAGTC | 1533 |
| ICE*Pmi*ChnHBSZC23、ICE*Pmi*ChnSCNNC24、ICE*Pmi*ChnHBSZC16 | Sul2-tnpA-F | CAGGAAAGCGGCCTATGGAG | 1574 |
|  | Sul2-tnpA-R | GCCTTTACCGGCCTCACATT | 1574 |
|  | LysR-strB-F | TTGGTATCAGCCAGTCGAGC | 1533 |
|  | LysR-strB-R | ACTCCTGCAATCGTCAAGGG | 1533 |
|  | tn3-tnpA-F | GTCCAGCAAGCCTTGAACAG | 1544 |
|  | tn3-tnpA-R | ACCGAATGCGATTTCCGTCT | 1544 |
|  | TnpA-tnpB-F | TGCGGTTACAGTTTTGCACC | 1519 |
|  | TnpA-tnpB-R | GCACCCACCCCATGATCTC | 1519 |

**continued Table 1：**

| **Strain** | **Primer** | **Sequence** | **Lenth（bp）** |
| --- | --- | --- | --- |
| ICE*Pmi*ChnSCSZC17、ICE*Pmi*ChnSCSZC25 | TnpB-F | GAATTTCTCCAATGCGGGCG | 1507 |
|  | TnpB-R | TTGGACCGCAGTTGACTCTT | 1507 |
|  | LysR-tnpB-F | CTGGCAGGATACCGCTCATT | 1511 |
|  | LysR-tnpB-R | TGCTCGGTCGTGAGAACAAT | 1511 |
|  | Dns-tnpA-F | TGGCAGTTGCCGACTATCAG | 1500 |
|  | Dns-tnpA-R | TCGAGATCGTCAGCATGGAC | 1500 |
|  | TR-tnp26-F | CCTTGGTAAATCGCTGCGTG | 1531 |
|  | TR-tnp26-R | GCCTCGGTGAGTTTTCTCCT | 1531 |
|  | AphA1-tnp26-F | CTTGATGGTCGGAAGAGGCA | 1508 |
|  | AphA1-tnp26-R | CATGCGGATCAGTGAGGGTT | 1508 |
|  | AadA2-tnpA-F | TTATCCGGCTAAGCGCGAG | 1511 |
|  | AadA2-tnpA-R | TCCTGGGCATTGACGAGCTA | 1511 |
|  | Hp-s063-F | TTCAGTTTCGAGGTGACGGG | 1520 |
|  | Hp-s063-R | CGTGTCGTTGACCGTGTTTT | 1520 |
| ICE*Pmi*ChnSCDJC2、ICE*Pmi*ChnHBSZC16、ICE*Pmi*ChnHBNNC12、ICE*Pmi*ChnHBRJC2、ICE*Pmi*ChnSCRJC3、ICE*Pmi*ChnSCBC11-9、ICE*Pmi*ChnSCRJC4、ICE*Pmi*ChnSCRJC5 | TnpA-B-F | TCAGTTCCGTGGCAAAAACG | 1538 |
|  | TnpA-B-R | AAGTGAACACCTTTCGCCCC | 1538 |
|  | TnpB-hp-F | GCCTTGCTGGTTGATGAAGT | 1501 |
|  | TnpB-hp-R | CGATCCGCTCGACGATACC | 1501 |
|  | LysR-F | GTCGCCGAGCATCTGAATTT | 208 |
|  | LysR-R | TCTTCACTGCGTGATCGAGTT | 208 |
|  | Tran-F | TTCTCACGAGAGCAGAACAGG | 349 |
|  | Tran-R | AAGTGCTGACTTTCGTCCCAT | 349 |
|  | Chn1-tnpA-F | GTTCACCTCGTTTCGGTTGC | 1546 |
|  | Chn1-tnpA-R | CATGACCGAACTTCCCGACA | 1546 |
|  | MerR-tetA-F | GATCGGCAGGACAACGGTTA | 1418 |
|  | MerR-tetA-R | AACCAACCCTTGGCAGAACA | 1418 |
|  | TetR-tnpA-F | TCAATTGCTTACCGGGCCTT | 1427 |
|  | TetR-tnpA-R | TTAGCGATGAGGCAGCCTTT | 1427 |
|  | TnpA-iroN-F | AACGCGGAGTGAATGTCGAT | 1408 |
|  | TnpA-iroN-R | GCTCCAGCCTATACGGTGAC | 1408 |

**continued Table 2：**

| **Strain** | **Primer** | **Sequence** | **Lenth（bp）** |
| --- | --- | --- | --- |
|  | TnpA-chrA-F | CCAGCGGTAAATCGTGGAGT | 1454 |
|  | TnpA-chrA-R | TGCGGCTTGGATTGACATCT | 1454 |
|  | chrA-TnpA-F | GCTTGGATGCCTGTATTGCG | 1485 |
|  | chrA-TnpA-R | ATGTCACGCTGAAAATGCCG | 1485 |
|  | TnpA5-blmS-F | ACGGCATCAGTTACCGTGAG | 1526 |
|  | TnpA5-blmS-R | TGCAGCCCAAGTGTAGAATG | 1526 |
|  | NimC-aac-F | CGGTGAGCCGAATCCATTTTC | 1541 |
|  | NimC-aac-R | TCGGTTTCTTCTTCCCACCG | 1541 |
|  | Arr3-qacEdelta1-F | TTTGGCGATTGGTGACTTGC | 792 |
|  | Arr3-qacEdelta1-R | AGTCCCGACCAGACTGCATA | 792 |
|  | Sul1-tnpA-F | ATCAGACGTCGTGGATGTCG | 1486 |
|  | Sul1-tnpA-R | AACGCGGAGTGAATGTCGAT | 1486 |
|  | TnpA8-TnpA-F | TAGTGCACGCATCACCTCAA | 1542 |
|  | TnpA8-TnpA-R | GTCGTAGATGCGCCGGTTAG | 1542 |
|  | TnpA9-pm-F | CAACATTCACACCCCGTTCG | 1507 |
|  | TnpA9-pm-R | TCGACCTTATGGTGCGCAAA | 1507 |
|  | Sul2-dmpL-F | ATGCCGGGATCAAGGACAAG | 1025 |
|  | Sul2-dmpL-R | AGGGCCGTACTGTAATCCAA | 1025 |
|  | AacC4-tnpA-F | TCAACCGAGCAAAGGCATGA | 1459 |
|  | AacC4-tnpA-R | CTCTGCTTACCAGGCGCATT | 1459 |
|  | TnpA-intL1-F | TGACATCATTCTGTGGGCCG | 1458 |
|  | TnpA-intL1-R | GCGCATACGCTACTTGCATT | 1458 |
|  | aadA2-tnpA-F | CGCATTTGGTACAGCGCAAT | 609 |
|  | aadA2-tnpA-R | GGAGTCCATCAACCGACTGG | 609 |
|  | MerR-hp-F | AATCCGATGAGATGCCGGTC | 707 |
|  | MerR-hp-R | CAGTGCGCTTTACGGAATCG | 707 |
